# Characterization and Inhibitory Effects of Essential Oil and Nanoemulsion from *Ocotea indecora* (Shott) Mez in *Aspergillus* Species

**DOI:** 10.3390/molecules28083437

**Published:** 2023-04-13

**Authors:** Leonardo de Assunção Pinto, Francisco Paiva Machado, Ricardo Esteves, Victor Moebus Farias, Felipe Braz Nielsen Köptcke, Eduardo Ricci-Junior, Leandro Rocha, Luiz Antonio Moura Keller

**Affiliations:** 1 Programa de Pós-Graduação em Biotecnologia Vegetal e Bioprocessos, Centro de Ciências em Saúde, Universidade Federal do Rio de Janeiro, Rio de Janeiro CEP 21941-590, Brazil; fpmachado@ufrj.br (F.P.M.); ricardogdb@gmail.com (R.E.); 2 Programa de Pós-Graduação em Higiene Veterinária e Processamento Tecnológico de Produtos de Origem Animal, Faculdade de Veterinária, Universidade Federal Fluminense, Niterói, Rio de Janeiro CEP 24220-000, Brazil; victormoebus@id.uff.br; 3 Faculdade de Farmácia, Universidade Federal Fluminense, Niterói, Rio de Janeiro CEP 24241-000, Brazil; felipekoptcke@id.uff.br (F.B.N.K.); leandromr@id.uff.br (L.R.); 4 Departamento de Medicamentos, Faculdade de Farmácia, Universidade Federal do Rio de Janeiro, Rio de Janeiro CEP 21941-902, Brazil; ricci@pharma.ufrj.br; 5 Laboratório de Tecnologia de Produtos Naturais, Faculdade de Farmácia, Universidade Federal Fluminense, Niterói, Rio de Janeiro CEP 24241-002, Brazil; 6 Departamento de Zootecnia e Desenvolvimento Agrosustentável, Faculdade de Veterinária, Universidade Federal Fluminense, Niterói, Rio de Janeiro CEP 24220-000, Brazil; luiz_keller@id.uff.br

**Keywords:** Flavi section, fungistatic, aflatoxin, natural products, sesquirosefuran, sesquiterpenes

## Abstract

The *Aspergillus* genus, the etiological agent of aspergillosis, is an important food contaminant and mycotoxin producer. Plant extracts and essential oils are a source of bioactive substances with antimicrobial potential that can be used instead of synthetic food preservatives. Species from the Lauraceae family and the *Ocotea* genus have been used as traditional medicinal herbs. Their essential oils can be nanoemulsified to enhance their stability and bioavailability and increase their use. Therefore, this study sought to prepare and characterize both nanoemulsion and essential oil from the *Ocotea indecora*’s leaves, a native and endemic species from the Mata Atlântica forest in Brazil, and evaluate the activity against *Aspergillus flavus* RC 2054, *Aspergillus parasiticus* NRRL 2999, and *Aspergillus westerdjikiae* NRRL 3174. The products were added to Sabouraud Dextrose Agar at concentrations of 256, 512, 1024, 2048, and 4096 µg/mL. The strains were inoculated and incubated for up to 96 h with two daily measurements. The results did not show fungicidal activity under these conditions. A fungistatic effect, however, was observed. The nanoemulsion decreased the fungistatic concentration of the essential oil more than ten times, mainly in *A. westerdjikiae*. There were no significant changes in aflatoxin production.

## 1. Introduction

One of the most critical problems reported in human history is hunger. Even with all the breakthroughs and innovations since the implementation of agriculture, it was not enough to free us from this malady. In 2022, almost 830 million people, about 10% of the global population, suffered from hunger [1].

The Food and Agriculture Organization (FAO) estimates that up to 25% of the world’s cereal grains are contaminated by fungi and/or mycotoxins in the field or during storage [2,3], leading to a mass wastage of food.

Members of the genus *Aspergillus* spp. have the strongest ecological link to the human food supply [4]. The genus presents a worldwide distribution, with 339 species [5], divided into sections and clades. The clade *A. flavus*, in section Flavi, is important for containing the most common agents of superficial and invasive aspergillosis [6].

The species of the *A. flavus* clade are still linked to the production of mycotoxins, secondary metabolites with harmful toxic effects on animals and humans [7]. The main mycotoxins produced by fungi of the genus *Aspergillus* spp. are aflatoxins (AF) and difuranocoumarins, with rigid and flat structures that form the four major substances: B1, B2, G1, and G2 [8]. This chemical stability gives them high resistance to heat treatments, extreme pH values, high pressures, and food-grade chemical treatments [9], making them also detectable at various levels of the production chain [7].

The ingestion of AFB1 can lead to acute conditions associated with cellular damage and metabolism disruption. Chronic conditions can cause damage to the metabolization organs, especially those related to nephrotoxicity and hepatotoxicity, including oncogenesis [10,11].

Such hazardous substance, as expected, has a strict limit for concentrations in each medium, particularly for food supplies. The limits are determined by legislation worldwide and vary for each country. The Brazilian legislation, aligned with the MERCOSUL designations, established similar limits to the most accepted international recommendations, the ones in the *Codex Alimentarius* [12]. The limits range from 5 to 20 µg/kg, except for those outlined for children, with a limit of 1 µg/kg [13].

Inhibiting the growth and mycotoxin production of the food-related strains, therefore, shows itself as an essential strategy to combat worldwide hunger and is a matter of public health. These issues interconnect with the United Nations 17 Sustainable Development Goals for 2030, principally goals 2 and 3, respectively, “Zero Hunger” and “Good Health and Well-Being” [14].

Fungal and aflatoxin contamination can occur before, during, or after harvest, especially during storage and processing. Methods for preventing contamination can be divided into pre-harvest, harvest, and post-harvest strategies [15].

Pre-harvest factors include seed and cultivation conditions and the prevention of fungal infestations, considering environmental factors that influence infection [16]. Most of the threats, however, are post-harvest factors. These range from harvesting patterns to transport to the consumer, with a particular focus on storage [15]. About 25% of harvested fruits and vegetables are lost due to diseases, mainly caused by fungi. Although more severe in developing countries, it is not insignificant in developed countries, as the annual economic loss of the United States is around US$ 1 billion [16].

The usual method to prevent fungal contaminations in pre- and post-harvest is using chemical additives. The environmental and human health impact and the possible development of new resistant strains have forced the industry to seek new strategies as “clean label” alternatives [17].

Products extracted from plants and their formulations are classified mainly by the Food and Drug Administration (FDA) as Generally Recognized as Safe (GRAS) [18]. These products can be used in Europe and the USA as antimicrobial agents in pre- and post-harvest and food and feed additives, with a wide acceptance by consumers [19].

Among the plant-based products that can be obtained, essential oils (EO) are an option with proven methodologies and recognized potential, aiming to isolate the volatile chemicals found in low concentrations in plants. EOs stand out among plant derivatives because they often present biological activity [20]. In addition, they can be used in formulations such as nanoemulsions (NE), that may enhance their properties.

Nanoemulsions, like traditional emulsions, are dispersions of one immiscible liquid in another. Both are kinetically stable, but thermodynamically unstable systems. To reduce the natural tendency of phase separation by creaming, flocculation, coalescence, or Ostwald ripening, surfactants are ordinarily used to decrease the interfacial tension and improve the long-term stability of the nanodispersions [21].

The principal difference between nanoemulsion and traditional emulsion is that nanoemulsion droplets are on a nanometer scale, between 20 and 200 nm. Another difference is the translucid monophasic appearance, often with the bluish color of the Tyndall optical effect of light dispersion [22].

The conventional applicability of nanoemulsions is to enable oily phases, such as essential oils, in aqueous matrices. Recently, nanoemulsified essential oils have been spotlighted for developing new antimicrobial products [23]. They are a promising tool for antimicrobial delivery, and among the advantages are bioavailability enhancement, increased stability, a larger surface area, and improved bioactivity, allowing more effective interactions with microorganisms [24,25].

*Ocotea indecora* (Lauraceae) is a native endemic plant from Brazil. Species from this family and genus have been used as traditional medicinal herbs. Regarding *Ocotea* genus, they present several groups of secondary metabolites recognized in the literature, especially steroids, terpenoids, and alkaloids [26]. This plant species is found in the remaining Mata Atlântica forest in Brazil’s south and southeast regions, especially in the sandbank areas of Rio de Janeiro state [27]. However, the coastal ecosystem where most of the current population resides is the highest deforested biome in the country, having less than 7% of its original area [28]. The devastation associated with the endemic nature of the species highlights the urgency and importance of its study.

The main metabolite related to the *Ocotea indecora*’s essential oil from leaves is the furanosesquiterpene sesquirosefuran. Even though most of the reports are about insecticidal effects [29,30,31], furanosesquiterpenes have several activities recognized in the literature as antioxidant, herbicidal, antibacterial, chemo-preventive agents, and principally for this study, antifungal [32,33]. Thus, essential oils and their possible effects are important research proposals for the biotechnological development of sustainable products.

The objectives of this work were to evaluate the effectiveness of the *O. indecora* leaves’ essential oil and nanoemulsion on the growth of *A. flavus* (RC 2054), *A. parasiticus* (NRRL 2999), and *A. westerdjikiae* (NRRL 3174), and the inhibition of aflatoxins.

## 2. Results

### 2.1. Essential Oil Extraction and Chemical Characterization

The EO yielded 0.4% and allowed the identification of three substances, totaling 91.93% of the oil (Table 1). The sesquirosefuran (86.13%) was widely predominant, followed by the sesquiterpenes hydrocarbons: (Z)-β-farnesene (3.33%) and allo-aromadendrene (1.55%).

### 2.2. Nanoemulsion Preparation, Characterization, and Thermal Stress Stability

**Figure 1:**
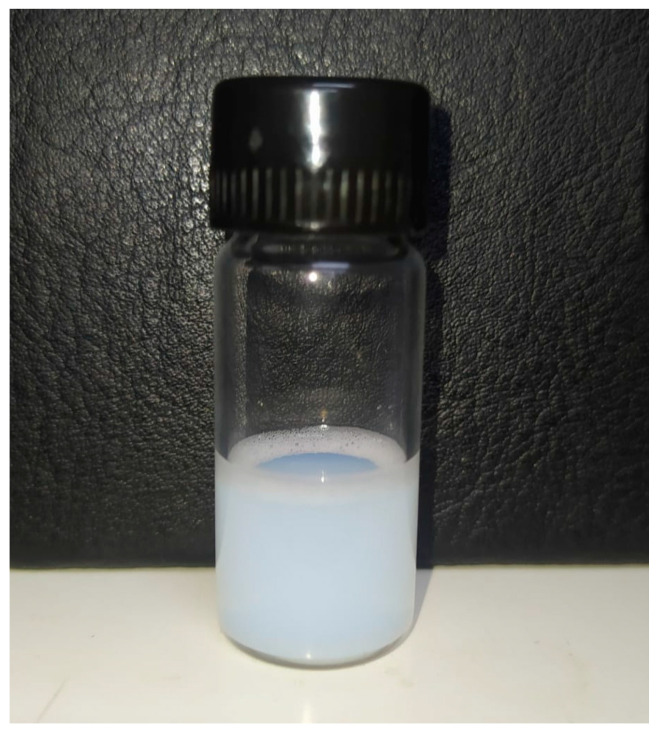
Nanoemulsion: bluish-white color characteristic of the Tyndall effect with 2% of NE.

**Figure 2:**
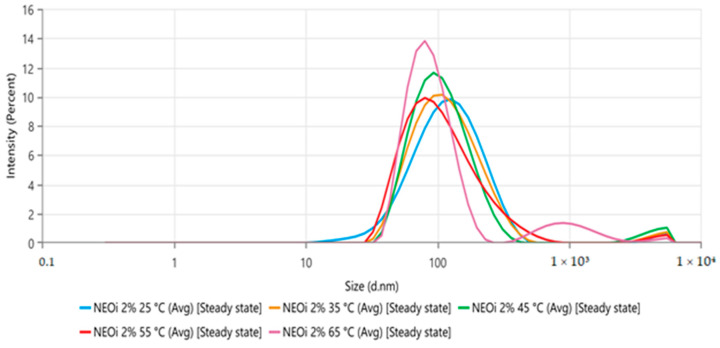
NE thermal stress (25–65 °C) size distribution by intensity.

The NE presented a white-bluish coloration (Figure 1), and the dynamic light scattering (DLS) analysis showed a mean droplet size (nm) of 103.4 ± 0.9. The polydispersity index (PdI) was 0.268 ± 0.010, and the zeta potential was −32.83 ± 0.8208 after preparation at room temperature (25 °C). The thermal stress stability of the nanoemulsion was realized initially at room temperature (25 °C) and increased to 65 °C at 10 °C/analysis. The size distribution by intensity represented in Figure 2 shows no relevant alterations in the nanoemulsion’s stability, while the droplet size and PdI values at each temperature can be seen in Table 2, showing statistical differences above 35 °C. After the thermal stress, no macroscopic changes were observed and the droplet size was 89.38 ± 2.153, the PdI was 0.246 ± 0.003, and the zeta potential was −34.85 ± 0.5284.

### 2.3. Inhibitory Effects on Aspergillus Strains

#### 2.3.1. *Aspergillus flavus*

The effects of the EO at 4096 µg/mL and the NE at 256, 512, 1024, and 2048 µg/mL on the growth of the *A. flavus* strain are presented in Table 3.

The colonies developed in the presence of the EO and the NE, demonstrating no fungicidal activity at the tested concentrations. The EO also showed no significant reduction (*p* > 0.05) compared to the control groups, evidencing no fungistatic activity.

As seen in Table 3, there was only a significant reduction up to 48 h (df = 5; F = 4.11; *p* = 0.021), but only for the NE in the concentration of 2048 µg/mL (*p* = 0.020). The statistical analysis for the 48 h incubation is shown in Table 4. No statistical significance was observed for the 72 h (*p* = 0.210) and 96 h (*p* = 0.135) incubations.

**Figure 3:**
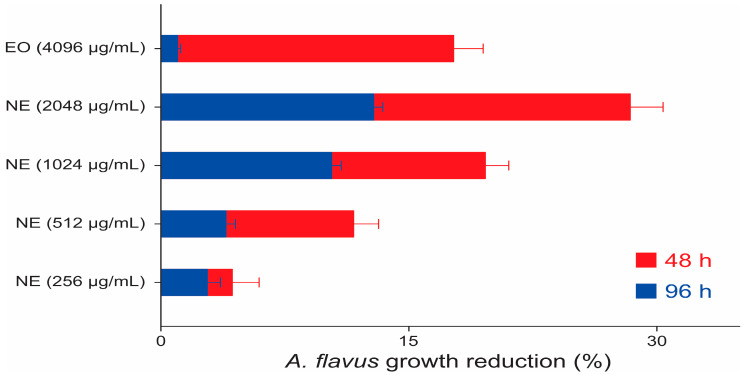
Inhibition of the *A. flavus* colonies by the concentrations of EO and NE at 48 and 96 h.

The percentages of reductions are shown in Figure 3, demonstrating the intensity and duration of the effects correlated to the tested concentrations. The highlight is the only significant inhibition that reached a 28.2% reduction in the colony diameter.

**Figure 4:**
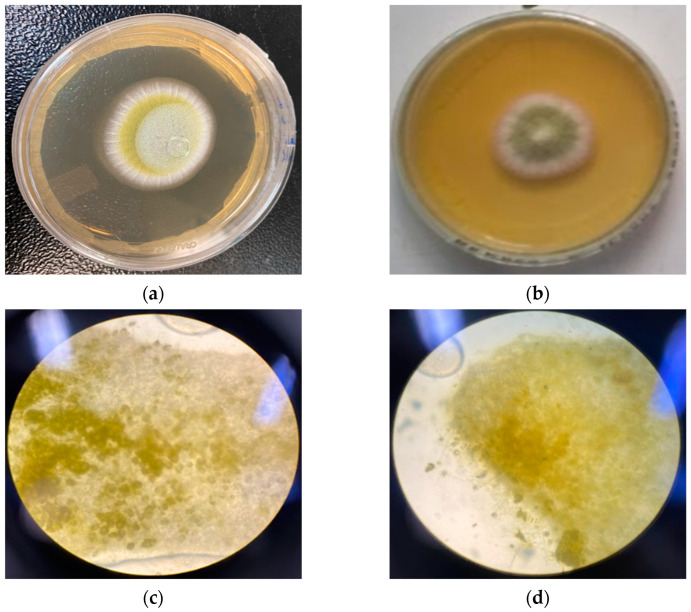
Macroscopic and microscopic comparison between the control colony and the colony with the NE at 2048 µg/mL at 48 h: (**a**) *A. flavus* colony, (**b**) *A. flavus* colony in the presence of the NE at 2048 µg/mL, (**c**) microscopy of *A. flavus*, and (**d**) microscopy of *A. flavus* in the presence of the NE at 2048 µg/mL.

Figure 4 compares the control colony and the colony with NE at 2048 µg/mL, showing a significant result. The photos were taken with a 48 h growth time and no difference was observed in either the macroscopic or microscopic morphology.

The mycotoxicological analysis, on average, showed that the production in all concentrations of the bioproduct did not show a difference with the control groups. The results are shown in Table 5.

#### 2.3.2. *Aspergillus parasiticus*

The trials’ results are shown in Table 6, demonstrating the effects of the EO at 4096 µg/mL and the NE at 256, 512, 1024, and 2048 µg/mL on the *A. parasiticus*’ growth.

The colonies showed growth in the presence of the EO at 4096 µg/mL, and compared to the control groups, showed no significant difference (*p* > 0.05). This demonstrates the essential oil concentration’s lack of fungicidal and fungistatic activity.

The growth of colonies in the presence of the NE showed no fungicidal activity and no significant effects were observed at both 72 h (*p* = 0.093) and 96 h (*p* = 0.164) of incubation. However, inhibition was observed in the first 48 h (df = 5; F = 9.96; *p* < 0.001).

While the NE concentrations of 256 and 512 µg/mL showed no effects even at 48 h, in the 1024 and 2048 µg/mL concentrations, a significant reduction in colonies’ growth was observed in the timeframe (*p* = 0.003 and *p* < 0.001, respectively), as shown in Table 7.

**Table 7:** One-way ANOVA between *A. parasiticus* colonies after 48 h of incubation with the EO and the NE.

Comparison	Diff of Means	*p*	q	*p*
Control 48 h vs. EOOi	0.392	6	2.932	0.361
Control 48 h vs. 2048 ppm	1.017	6	8.627	<0.001 *
Control 48 h vs. 1024 ppm	0.844	6	7.159	0.003 *
Control 48 h vs. 512 ppm	0.333	6	2.827	0.396
Control 48 h vs. 256 ppm	0.280	6	2.373	0.569

**Figure 5:**
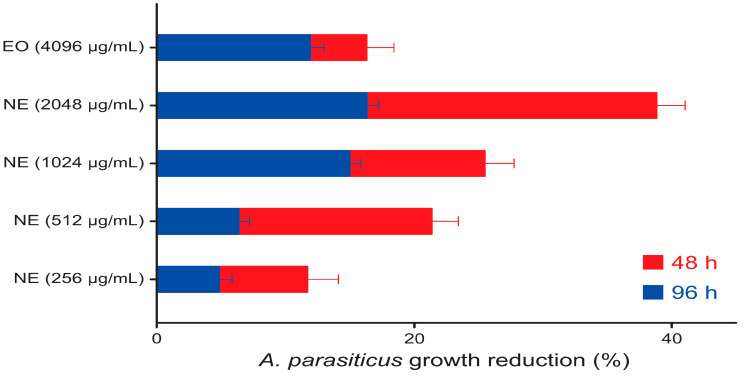
Inhibition of the *A. parasiticus* colonies by a concentration of the EO and the NE at 48 and 96 h.

* The values were recognized as statistically significant at a confidence interval of 95%. The duration and intensity of the effects are shown in Figure 5, especially the NE at 1024 µg/mL and 2048 µg/mL, which reached, respectively, 25.5% and 38.9% of inhibition.

**Figure 6:**
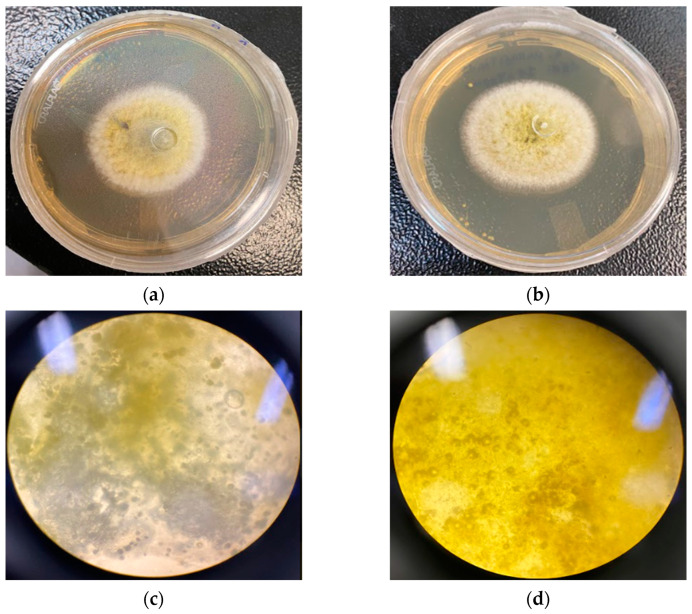
Macroscopic and microscopic comparison between the control colony and the colony with the NE at 2048 µg/mL at 48 h: (**a**) *A. parasiticus* colony, (**b**) *A. parasiticus* colony in the presence of the NE at 2048 µg/mL, (**c**) microscopy of *A. parasiticus*, and (**d**) microscopy of *A. parasiticus* in the presence of the NE at 2048 µg/mL.

The macroscopic and microscopic comparisons of the control colonies with the colonies in the presence of the NE at 2048 µg/mL at 48 h are shown in Figure 6, but no relevant morphological differences could be seen.

The AFB1 detection for all concentrations of the bioproduct again showed no activity compared to the controls. The quantification is shown in Table 8.

#### 2.3.3. *Aspergillus westerdjikiae*

Table 9 shows the effects on the growth of the *A. westerdjikiae* strain caused by the EO at 4096 µg/mL and the NE at 256, 512, 1024, and 2048 µg/mL.

The growth of the control groups and the EO at 4096 µg/mL showed no significant difference (*p* > 0.05) in the tested strain, indicating no fungicidal or fungistatic effect caused by the concentration of the tested EO. The NE in all concentrations also demonstrated a lack of fungicidal activity, but it did show fungistatic activity, as seen in Table 10.

**Figure 7:**
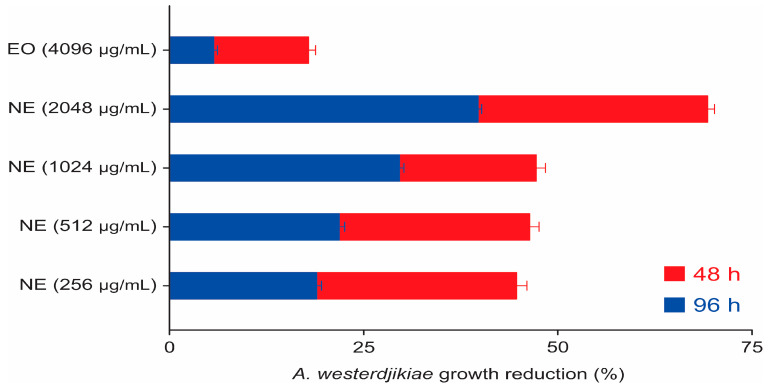
Inhibition of the *A. westerdijikiae* colonies by a concentration of EO and NE at 48 and 96 h.

In the first 48 h, all concentrations led to a significant reduction (df = 5; F = 9.53; *p* < 0.001), approximately 44% to 48%, except the concentration of 2048 µg/mL that reached 69% inhibition. The reduction by NE at 512 and 256 µg/mL decreased to nearly 20% after 48 h, while the 1024 µg/mL and 2048 µg/mL concentrations remained significant at 72 h (df = 5; F = 6.28; *p* = 0.004) and 96 h (df = 5; F = 7.03; *p* = 0.003), when they decreased to, respectively, 29.6% and 39.7% inhibition. All the reductions are graphically demonstrated in Figure 7.

**Figure 8:**
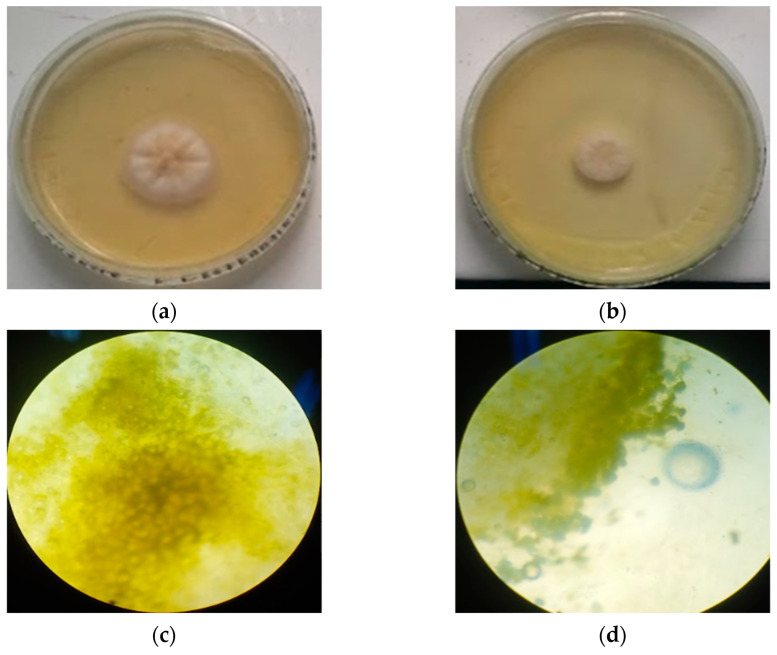
Macroscopic and microscopic comparison between the colonies with or without the NE at 2048 µg/mL at 48 h: (**a**) *A. westerdjikiae* colony, (**b**) *A. westerdjikiae* colony in the presence of the NE at 2048 µg/mL, (**c**) microscopy of *A. westerdjikiae*, and (**d**) microscopy of *A. westerdjikiae* in the presence of the NE at 2048 µg/mL.

The photos in Figure 8 compare the colonies after 48 h of incubation in the presence of the NE at 2048 µg/mL with the control colonies. The macroscopic and microscopic morphological comparison showed no observable difference.

As for the production of mycotoxins, the average of each strain in the presence of the products had no observable effects compared to the control production. The analysis outcome is demonstrated in Table 11.

## 3. Discussion

The essential oil from the leaves of *O. indecora* showed approximately 86% of sesquirosefuran, corroborating with the *O. indecora* chemical profile and sesquirosefuran amount (88–92%) described previously by other authors [29,31].

The sesquirosefuran is a furanosesquiterpene registered under CAS number 39007-93-7, with the molecular formula C_15_H_22_O and a molecular weight of around 218 u [36]. It has a log *p* of 5.847 and a TPSA equal to 13.140; then, according to the Pfizer Rule, it is likely to be toxic, especially as an inhibitor of some CYP family enzymes and a probable human hepatotoxic substance. The sesquirosefuran, however, is unlikely to cause severe skin, respiratory, and eye damage [37].

In this way, it is possible that the bioproduct can be used as an external preservative, as long as it does not involve direct consumption, and reinforcing that it is important to choose carefully where to use it in order not to cause unwanted damage to fauna, flora, and workers.

The sesquirosefuran has few reports about the molecule bioactivity, mainly insecticide activity [29,31], and none related to its antifungal potential. Sesquiterpenes and furanosesquiterpenes, in turn, have several recognized activities, and among them, Marongiu et al. [33] demonstrated activity against several *Aspergillus* sp. by essential oils rich in furanosesquiterpenes.

The furansesquiterpenes action mechanisms are not yet fully elucidated, but several studies describe antifungal effects related to the furan group in natural molecules [38,39]. Loi et al. [40], however, describe sesquiterpenes isolated from different plants capable of altering the mitochondrial function of mammals. The mitochondrial membrane potential is maintained in healthy individuals by an electrochemical gradient. The mechanism of action is not clearly understood, but it is theorized that a disturbance in the protons of the osmotic balance affects the electrochemical potential. As ATP levels decrease, metabolism and functions reduce until cell death, thus explaining the observed fungistatic effect.

The effect observed in mammal mitochondria is possibly similar in fungal mitochondria, as Campbell et al. [41] related similarities between both. This would explain the fungistatic effect and the absence of morphological alteration, as the inhibition could be a decrease in the metabolism caused only by the reduction in the ATP concentration.

Despite this report, *O. indecora*’s EO did not demonstrate a fungicidal or fungistatic effect at 4096 µg/mL and could not inhibit the aflatoxin production in the three *Aspergillus* strains tested. However, when the EO is nanoemulsified, the results are promising. The NE at 2048 µg/mL showed a fungistatic effect in the three strains, at least at 48 h, while the EO at a concentration two times higher did not show the same capacity.

The production of the EO nanoemulsion is an exciting tool to enable these lipophilic matrices into viable products and has been widely used in pharmaceutical and food industries [23]. In this study, the *O. indecora*’s EO was nanoemlusified by a low-energy approach to maintain the chemical profile since it does not use heat treatment in the nanoemulsification process, which avoids thermal degradation and volatilization of the terpenoids present in the EO [42].

There are two prevailing forces crucial to the stability of nanoemulsified systems: the gravitational (droplet size and weight) and the electrostatic repulsion (zeta potential). Together, they influence the physicochemical maintenance of a nanodispersion’s collective parameters [21,43]. The prepared nanoemulsion showed a bluish-white appearance consistent with the Tyndall effect, a reduced droplet diameter of 103.4 ± 0.9, and a 0.268 ± 0.010 PdI. As for the surface charge, one of the parameters related to the nanodroplets’ stability, the NE zeta potential, was −32.83 ± 0.8208, indicating good short-term stability, with coulombic repulsion between the negatively charged nanodroplets, and in that way, favoring the droplets’ Brownian motion [43]. Due to all these parameters, the product was characterized as a conventional monodispersed nanoemulsion [22].

The different nanoemulsion physicochemical properties, such as the reduced droplet diameter, may justify the higher effect of the NE in comparison to the EO. The increased fungistatic effect may be associated with higher bioavailability of the substances present in the EO. Furthermore, the increase of hydrophilicity and the nanoscale of the particles can lead to higher dispersion and stability in the medium [24] and may facilitate the absorption and metabolization of the substances present in the bioproduct [25]. The activity of the EO can be optimized in this way, by increasing the contact between the metabolites in the essential oil and the fungal cells.

Comparing the reduction pattern, a dose-dependent response of the NE against the strains was observed (Figure 3, Figure 5 and Figure 7). The fungistatic effect for the *A. flavus* strain was not significant below 2048 µg/mL but became significant upon reaching this concentration. The turning point for the *A. parasiticus* strain was even lower, as at 1024 µg/mL it had already shown a significant difference.

The comparison was even wider for the *A. westerdjikiae* strain, as all concentrations showed significant activity in the first 48 h, and the higher ones up to 96 h. The greater susceptibility of *A. westerdjikiae* to bioproducts was already reported in other studies, such as those of Rodrigues et al. [44] and Schlösser and Prange [45].

Even though all concentrations of the NE were active against the *A. westerdjikiae* strain, the dose-dependent effect was still visible. The NE showed activity that the OE in a concentration 16 times higher did not, therefore endorsing the potential caused by the nanoemulsification process.

The observed correlation between dosage and effect, and the NE showing a better result than the EO, even though it was in lower concentrations, indicate how the formulation can trigger and potentiate effects, whether expected or not, as observed in the study by Do Carmo Silva [46].

However, despite the potential, no concentration of the NE showed fungicidal activity in any of the strains. A concentration higher than 2048 µg/mL may be necessary to increase the bioavailability of the molecule even more to cause a proper fungicidal effect.

Another limitation observed was the drastic decrease in activity after 48 h of incubation as, after this point, only a fungistatic effect was observed in the colonies of the *A. westerdjikiae* strain and only with the NE at 1024 and 2048 µg/mL. This behavior may be due to the consumption of all the present NE by the fungal colonies, and only the residual effect was observed afterward. Another possibility is degradation over time in the incubation temperature and/or medium.

However, in the thermal stress stability study of the *O. indecora* nanoemulsion, only temperatures from 35 °C slightly reduced the droplet size (nm). As the incubation temperature was 25 to 30 °C, it is unlikely that a significant degradation occurred. The droplet reduction is probably associated with the increased solubility of essential oil terpenoids due to the higher temperature in the aqueous phase [47].

Regarding the size homogeneity of the nanodroplets, there was no statistical significance (*p* < 0.05) in the polydispersity index between all temperatures analyzed (25 to 65 °C). The zeta potential after the thermal stress was −34.85 ± 0.5284, suggesting no relevant alteration in the nanodroplets’ surface charge [43]. Both parameters reassured the improbability of degradation.

While the bioproduct showed a short period of a fungistatic effect and no fungicidal effect, it was still significant. The activity was seen in the exponential phase of growth for the three strains and, even though it did not establish a stationary range, the reduction in the log phase points to a potential for concomitant use with other antifungal products. The promising potential in associated substances of plant origin and commercial products was also described by Chagas [48].

Another point worth noting is that high-complexity food matrices can lead to reduced effectiveness of antimicrobials. Therefore, a larger amount of preservative than the one used in vitro tends to be necessary to achieve the same results. Higher concentrations, however, can impair the organoleptic properties of foods. To avoid this problem, lower concentrations of bioproducts with fungistatic effects can be used [39]. This highlights the use of nanoemulsions as food preservatives, as long as they are obtained from safe plant derivatives, since the present work demonstrated that they have exactly this desired capacity.

As for the quantification of AF, the variations were considered insignificant, according to statistical results, regardless of the concentration. However, there are reports of the chemical properties of the AFs being modified in different ways after incubation with plant extracts, including removal of the furan double bond in AFB1 and modification of the lactone ring, resulting in a significant decrease in cytotoxicity and carcinogenicity [49].

About the variations, considering the high sensitivity of the AF production [50], the oscillation may be due to the alteration in the fungal metabolism and its metabolites [51].

When comparing the results obtained with other descriptions in the literature, it is observed that this is the first study to report the antifungal properties of *Ocotea indecora* and its major substance, sesquirosefuran. Other studies on the genus have been carried out, however, few species have a chemical composition similar to the *O. indecora*. Furthermore, most trials are on the clinically important leviduriform fungal species.

Focusing only on species with a high content of sesquiterpenes in their EO and their activities on filamentous fungi, the studies by Prieto et al. [52] stand out, where *Ocotea macrophylla* is observed with 70% of the constituents being sesquiterpenes, and this oil demonstrates antifungal activity against species of the genus *Fusarium*. Another significant trial is that carried out by Mezzomo et al. [53], who observed *Ocotea puberula*’s EO with 17% of sesquiterpenes and reported weak antifungal activity in two *Aspergillus* species, *alternate* and *flavus*.

The low antifungal effect was, therefore, concordant with the literature. However, the potentiation demonstrated by the nanoemulsification of the *O. indecora* essential oil qualifies the nanoemulsion as a fungistatic agent for the tested strains. Combined with their biodegradation and metabolization properties, the nanoemulsion was demonstrated as an appropriate option for pre-harvest treatment that, if well-used, will not compromise the environment or the workers’ and consumers’ health.

## 4. Materials and Methods

### 4.1. Plant Material

The fresh leaves of *O. indecora* were collected in the Restinga of Jurubatiba National Park, Carapebus, RJ, Brazil (“22°12.683′ S”, “41°35.283′ O”, “22°12.703′ S”, and “41°35.336′ O”). The obtention and research of the plant material were authorized by SisBio/ICMBio (13659-14) and SisGen (A0D648D). The species was identified, and a voucher specimen was deposited at Universidade Estadual do Rio de Janeiro—Faculdade de Formação de Professores (UERJ—FFP) herbarium, under registration number RFFP: 16.873.

### 4.2. Essential Oil Extraction and Chemical Characterization

Fresh leaves of *O. indecora* (250 g) were crushed into a blender with distilled water, transferred to a 5.0 L round-bottom flask, and subjected to hydrodistillation in a Clevenger-type apparatus for 4 h. After that, the essential oil was dried over anhydrous sodium sulfate and stored in an amber glass vial at 4 °C.

The essential oil was analyzed in a GC-MS QP2010 (Shimadzu) gas chromatograph equipment coupled with a mass spectrometer and a GC-2014 (Shimadzu) gas chromatograph equipped with a flame ionization detector (FID). The chromatographic conditions were: a 260 °C injector temperature, with the carrier gas helium, the flow rate was 1 mL/min, and the split ratio was 1:40. Initially, the oven temperature began at 60 °C and then increased to 290 °C (3 °C/min rate). The essential oil (1 µL) was dissolved in dichloromethane (1:100 μL) and injected into a DB-5 column for MS (0.25 mm ID, 30 m in length, 0.25 μm film thickness). The mass spectrometry conditions were 70 eV electron ionization and a 1 scan/s scan rate. The GC-FID analysis was similar to the MS, except for the injection in a DB-5 column (0.25 mm ID, 30 m in length, 0.25 μm film thickness) and the FID temperature at 290 °C. The arithmetic index (AI) was calculated by interpolating the retention times of a mixture of aliphatic hydrocarbons (C7–C40) and analyzed under the same chromatographic methods. Substances were identified by comparing their retention indices and mass spectra with those reported in the literature [34,35]. Compounds’ mass spectra fragmentation pattern was also compared with NIST mass spectrum libraries. GC-FID performed the relative abundance of the chemical constituents under the same conditions as GC-MS. The FID peak area normalization method obtained the percentages of these compounds.

### 4.3. Nanoemulsion Preparation, Characterization, and Thermal Stress Stability

The formulation of the nanoemulsion of *O. indecora* was previously described by Machado et al. [29]. The nanoemulsion was prepared by the low-energy method. The NE aqueous phase was 96% (*w/w*) of distilled water, and the oil phase was made up of 2% (*w/w*) of EO, and 2% (*w/w*) of the surfactants polysorbate 20 and sorbitan monooleate 80 in a 4:1 proportion, respectively. The oil phase was homogenized in a vortex for 1 min, and then the aqueous phase was slowly dripped into the oil phase in continuous agitation.

The droplet size (nm), zeta potential (ZP), and polydispersity index (PdI) from the NE diluted with distilled water (1:40) were characterized by dynamic light scattering (DLS) in a Zetasizer Advance Lab Blue (Malvern Instruments^®^, Worcestershire, UK).

The *O. indecora* nanoemulsion was submitted to thermal stress after preparation to assess the trend of the nanoemulsion droplet size, zeta potential, and polydispersity index with a temperature increment from 25 to 65 °C, with an increase of 10 °C between analyses. The characterization was realized under the same conditions described above.

### 4.4. Fungal Strains

The strains used were *A. flavus* RC 2054, *A. parasiticus* NRRL 2999, and *A. westerdjikiae* NRRL 3174, all reference strains known to produce aflatoxin B1 (AFB1).

### 4.5. Inoculation and Incubation Conditions

The methodology was adapted from Rodrigues et al. [54], with the direct addition of the bioproduct in the medium and then needle-inoculating the strains.

The EO samples were diluted with 10% dimethylsulfoxide (DMSO) and tested at a 4096 µg/mL concentration in the culture medium. As for the NE, the concentrations of 256, 512, 1024, and 2048 µg/mL were tested in the medium.

The medium used was Kasvi’s Sabouraud Dextrose Agar (SDA), with a standardized volume of 25 mL being used for each culture, thus allowing control of the study concentrations [55]. The EO and the NE samples were added to the SDA in sufficient volume to reach the determined concentrations.

The Petri plates with the bioproducts were needle-inoculated centrally with 10 µL of the spore suspension. The plates were incubated at 25–30 °C for 96 h, with daily observation and diameter measurement.

Plates containing SDA medium without essential oil were used as a control group (Control 1), and the diluents, DMSO, and a nanoemulsion without the EO were used as a second control group (Control 2).

### 4.6. Growth Assessment

The diameter of the growing colonies was measured daily for four days. The percentages of inhibition of diameter growth (PIDG) values were determined according to the equation below:

(1) $$\mathrm{PIDG}\left(\%\right)=100\times \frac{\left(\mathrm{Diameter}\;\mathrm{of}\;\mathrm{control}-\mathrm{Diameter}\;\mathrm{of}\;\mathrm{sample}\right)}{\mathrm{Diameter}\;\mathrm{of}\;\mathrm{control}}$$

### 4.7. Microscopic Evaluation

A 1 mm piece of the colonies was collected for each plate of the three strains for the observed points. The pieces were then placed in proper slides for the optical microscope with one drop of distilled water and then set with the coverslip. The slides were observed in a Nikon ALPHAPHOT-2 YS2-H at 1000× magnification.

### 4.8. Aflatoxin Analyzes

The detection and quantification of aflatoxins was performed based on Geissen [56], taking a three-point sample for each plate with fungal growth and placing it in microtubes in duplicates. Later, 0.5 mL of chloroform was added to each microtube and then centrifuged at 4000 rpm for 10 min. After precipitation, the extract was removed and transferred to another microtube to dry and the contents were resuspended with 70% methanol. The quantification was realized by HPLC.

### 4.9. Statistical Analyses

Data evaluations were performed by analysis of variance (ANOVA). Data were transformed using the logarithmic function, log10 (x + 1), before ANOVA, and when necessary, the data were transformed with a root square to obtain a homogeneous data distribution. Tukey’s test was used to compare the enumeration data of the different concentrations of the evaluated products, the different presentations of the tested products, and the variations in incubation and growth times. All analyses were based on evaluating these substances’ fungicidal or fungistatic potential. Analyses were conducted using the PROC GLM computer program in SAS (SAS Institute, Cary, NC, USA).

## 5. Conclusions

The *O. indecora* essential oil did not show fungicidal potential at the concentrations tested against *A. flavus* RC 2054, *A. parasiticus* NRRL 2999, and *A. westerdjikiae* NRRL 3174. However, the *O. indecora* nanoemulsion showed fungistatic potential in *Aspergillus* strains. At 256 and 512 µg/mL, the EO affected the growth of the *A. westerdjikiae* strain for 48 h. At 1024 µg/mL, the EO inhibited the growth in both *A. parasiticus* and *A. westerdjikiae* for 48 and 96 h. The higher concentration, 2048 µg/mL, showed activity in all three strains up to 48 h, and up to 96 h for the *A. westerdjikiae*.

The nanoemulsion of the *Ocotea indecora* can be considered a fungistatic agent for the tested strains, and the formulation of the essential oil in the nanoemulsion triggered and potentiated effects that otherwise would not be significant.

The aflatoxin evaluation indicated that the products in the tested concentration had no significant effect on the production of secondary metabolites by the studied strains.

The study indicated an exciting line of research not only in the prospection of natural products but also for the application of nanoemulsions as a bioactive carrier, especially those that have, by nature, hydrophobic properties, and the results should be applied to other promising plant derivatives.

## Figures and Tables

**Table 1:** Chemical characterization of the EO by GC-MS and GC-FID.

	RT	AI_exp_	AI	Substances	%
1	26.970	1448	1440	(Z)-β-Farnesene ^a^	3.33965
2	27.323	1457	1458	allo-Aromadendrene ^a^	1.55898
3	30.802	1545	1549	Sesquirosefuran ^b^	86.13331
Total identified					91.03194
Sesquiterpene hydrocarbons					4.89863
Sesquiterpene oxygenated					86.13331

RT: retention index; AI: arithmetic index; AI_exp_ arithmetic index calculated. ^a^ Identified from Adams [34]. ^b^ Identified from Pherobase (El- Sayed) [35].

**Table 2:** NE mean size and PdI values from the thermal stress stability study (25–65 °C).

Temperature (°C)	Average Size (nm)	Polydispersity Index
25	103.40 ± 0.90	0.239 ± 0.010
35	102.90 ± 0.519	0.247 ± 0.015
45	98.97 ± 0.312 *	0.241 ± 0.018
55	91.58 ± 0.515 *	0.252 ± 0.009
65	89.38 ± 2.15 *	0.246 ± 0.003

* The values were recognized as statistically significant at a confidence interval of 95%.

**Table 3:** Diameter (mm) of *A. flavus* colonies in the presence of EO and NE at 48, 72, and 96 h.

Bioproduct	48 h	72 h	96 h
Control	20.90 ± 1.838	32.75 ± 3.942	43.35 ± 3.470
EO 4096 µg/mL	17.20 ± 3.960	32.90 ± 0.990	42.30 ± 2.121
NE 256 µg/mL	20.00 ± 1.212	31.90 ± 2.402	43.30 ± 1.900
NE 512 µg/mL	18.50 ± 1.769	30.60 ± 1.200	42.30 ± 1.513
NE 1024 µg/mL	16.50 ± 0.854	28.50 ± 1.114	40.00 ± 1.682
NE 2048 µg/mL	15.00 ± 2.287 *	28.50 ± 2.921	37.80 ± 3.651

* The values were recognized as statistically significant at a confidence interval of 95%.

**Table 4:** One-way ANOVA between *A. flavus* colonies with or without the EO and the NE.

Comparison	Diff of Means	*p*	q	*p*
Control 48 h vs. EO	3.700	6	3.055	0.322
Control 48 h vs. 2048 ppm	5.900	6	5.524	0.020 *
Control 48 h vs. 1024 ppm	4.400	6	4.119	0.104
Control 48 h vs. 512 ppm	2.400	6	2.247	0.620
Control 48 h vs. 256 ppm	0.900	6	0.843	0.989

* The values were recognized as statistically significant at a confidence interval of 95%.

**Table 5:** AFB1 production by *A. flavus* colonies in the presence of EO and NE at 48, 72, and 96 h.

Bioproduct	Concentration *
Control	9.06 ± 1.50 µg/kg
EO 4096 µg/mL	9.81 ± 1.09 µg/kg
NE 256 µg/mL	9.79 ± 1.07 µg/kg
NE 512 µg/mL	9.55 ± 1.61 µg/kg
NE 1024 µg/mL	9.48 ± 1.72 µg/kg
NE 2048 µg/mL	9.38 ± 1.52 µg/kg

* No significant difference observed in the Tukey’s test (*p* > 0.05) for the different concentrations.

**Table 6:** Diameter (mm) of *A. parasiticus* colonies in the presence of the EO and the NE at 48, 72, and 96 h.

Bioproduct	48 h	72 h	96 h
Control	21.75 ± 2.453	35.60 ± 4.510	45.30 ± 6.056
EO 4096 µg/mL	18.20 ± 0.141	31.40 ± 2.828	41.30 ± 3.110
NE 256 µg/mL	19.20 ± 1.852	32.30 ± 3.676	43.10 ± 3.105
NE 512 µg/mL	17.10 ± 3.804	28.20 ± 4.518	42.40 ± 1.952
NE 1024 µg/mL	16.20 ± 1.735 *	28.10 ± 2.797	38.50 ± 2.835
NE 2048 µg/mL	13.30 ± 1.873 *	27.90 ± 3.579	37.90 ± 1.411

* The values were recognized as statistically significant at a confidence interval of 95%.

**Table 8:** AFB1 production by *A. parasiticus* colonies in the presence of EO and NE at 48, 72, and 96 h.

Bioproduct	Concentration *
Control	31.71 ± 4.67 µg/kg
EO 4096 µg/mL	36.24 ± 5.35 µg/kg
NE 256 µg/mL	33.81 ± 4.28 µg/kg
NE 512 µg/mL	33.67 ± 3.64 µg/kg
NE 1024 µg/mL	31.88 ± 2.36 µg/kg
NE 2048 µg/mL	30.14 ± 3.64 µg/kg

* No significant difference observed in the Tukey’s test (*p* > 0.05) for the different concentrations.

**Table 9:** Diameter (mm) of *A. westerdjikiae* colonies in the presence of the EO and the NE at 48, 72, and 96 h.

Bioproduct	48 h	72 h	96 h
Control	11.90 ± 0.779	20.20 ± 1.922	31.00 ± 1.858
EO 4096 µg/mL	9.80 ± 0.990	20.20 ± 1.556	29.20 ± 1.273
NE 256 µg/mL	6.60 ± 1.735 *	16.50 ± 2.227	25.10 ± 3.509
NE 512 µg/mL	6.40 ± 1.682 *	16.30 ± 1.744	24.20 ± 3.064
NE 1024 µg/mL	6.30 ± 1.539 *	15.60 ± 1.800 *	21.80 ± 2.987 *
NE 2048 µg/mL	3.70 ± 2.894 *	11.20 ± 3.764 *	18.70 ± 4.414 *

* The values were recognized as statistically significant at a confidence interval of 95%.

**Table 10:** One-way ANOVA between *A. westerdjikiae* colonies with the EO and the NE in all incubation periods.

Comparison	48 h				72 h				96 h			
	Diff of Means	*p*	q	*p*	Diff of Means	*p*	q	*p*	Diff of Means	*p*	q	*p*
Control vs. EOOi	2.100	6	1.989	0.723	3.553 × 10^−15^	6	2.506 × 10^−15^	1.000	1.775	6	0.947	0.982
Control vs. 2048 ppm	8.200	6	8.805	<0.001 *	9.000	6	7.198	0.003 *	12.275	6	7.422	0.002 *
Control vs. 1024 ppm	5.600	6	6.013	0.011 *	4.600	6	3.679	0.035 *	9.175	6	5.548	0.019 *
Control vs. 512 ppm	5.500	6	5.906	0.013 *	3.900	6	3.119	0.303	6.775	6	4.097	0.107
Control vs. 256 ppm	5.300	6	5.691	0.016 *	3.700	6	2.959	0.352	5.875	6	3.552	0.195

* The values were recognized as statistically significant at a confidence interval of 95%.

**Table 11:** AFB1 production by *A. westerdjikiae* colonies in the presence of EO and NE at 48, 72, and 96 h.

Bioproduct	Concentration *
Control	15.79 ± 3.18 µg/kg
EO 4096 µg/mL	16.09 ± 2.86 µg/kg
NE 256 µg/mL	15.59 ± 2.54 µg/kg
NE 512 µg/mL	14.83 ± 2.73 µg/kg
NE 1024 µg/mL	13.92 ± 2.54 µg/kg
NE 2048 µg/mL	13.52 ± 2.48 µg/kg

* No significant difference observed in the Tukey’s test (*p* > 0.05) for the different concentrations.

## Data Availability

Not applicable.

## Sample Availability

Samples of the compounds are not available from the authors.
